# Protein phase separation hotspots at the presynapse

**DOI:** 10.1098/rsob.210334

**Published:** 2022-02-09

**Authors:** Janin Lautenschläger

**Affiliations:** Department of Clinical Neurosciences, Cambridge Institute for Medical Research, University of Cambridge, Cambridge CB2 0XY, UK

**Keywords:** synapse, phase separation, active zone, clathrin-mediated endocytosis, exocytosis, synapsin

## Abstract

Fundamental discoveries have shaped our molecular understanding of presynaptic processes, such as neurotransmitter release, active zone organization and mechanisms of synaptic vesicle (SV) recycling. However, certain regulatory steps still remain incompletely understood. Protein liquid–liquid phase separation (LLPS) and its role in SV clustering and active zone regulation now introduce a new perception of how the presynapse and its different compartments are organized. This article highlights the newly emerging concept of LLPS at the synapse, providing a systematic overview on LLPS tendencies of over 500 presynaptic proteins, spotlighting individual proteins and discussing recent progress in the field. Newly discovered LLPS systems like ELKS/liprin-alpha and Eps15/FCho are put into context, and further LLPS candidate proteins, including epsin1, dynamin, synaptojanin, complexin and rabphilin-3A, are highlighted.

## Graphical abstract

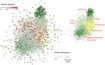

## 1. Introduction

When the first study demonstrated that proteins with low-complexity domains could undergo phase transition forming jelly-like hydrogels, this unveiled a new paradigm for the formation of subcellular compartments [1]. Nowadays, vast evidence emphasizes a role of protein phase separation in the formation of dynamic organelles like RNP transport granules, stress granules and P-bodies or in the compartmentalization of the nucleus [2,3]. More and more membrane-less compartments are identified, and the understanding that also the presynapse is sub-compartmentalized via liquid–liquid phase separation (LLPS) is emerging. The very first example of LLPS at the presynapse was synapsin-1 phase separation, shown to regulate synaptic vesicle (SV) clustering [4]. Furthermore, RIM/RIMBP LLPS has been demonstrated to couple local calcium entry to SV release at the active zone [5].

A protein's tendency to undergo protein phase separation is conveyed by amino acid regions that do not adopt a well-defined structure and are therefore called intrinsically disordered regions. These protein regions, until now, were more or less neglected for understanding a protein's function because no clear role could be assigned. However, it now becomes critical to understand which proteins or protein regions could participate in various LLPS systems. In addition, also protein regions with a less complex amino acid composition, called low-complexity domains, or protein regions with an enrichment of certain amino acids (i.e. proline, arginine, serine) tend to support weak protein–protein interactions (PPIs), known to be involved in LLPS.

To date, we have gained an initial understanding of presynaptic LLPS from recent studies; however, to understand this system in its full complexity we do need a better overall picture of which processes and proteins could be regulated and influenced by LLPS. Therefore, this article contains the first systematic analysis evaluating over 500 synaptic proteins for their intrinsic disorder, low complexity and enrichment of amino acids. It provides a novel resource and discusses known and potential presynaptic LLPS systems.

## 2. Methodology

This study followed a three-step approach. Presynaptic proteins were identified via UniProtKB and Gene Ontology [6–9]. IUPred2A, SMART and PAXdb were used to retrieve information about intrinsic disorder, low complexity domains, amino acid composition and protein abundance [10–12]. Protein-protein interaction profiles were obtained from the STRING database; Cytoscape and ClusterONE were used to perform PPI network analysis [13–15] (table 1).

**Table 1.** Data resources.

database	description	application	website
UniProtKB	The Universal Protein Resource (UniProt) is a comprehensive resource for protein sequence and annotation data. The UniProt databases are the UniProt Knowledgebase (UniProtKB), the UniProt Reference Clusters (UniRef) and the UniProt Archive (UniParc).	identification of relevant human proteins	https://www.uniprot.org/
		extraction of basic protein parameters	
IUPred2A	IUPred2A is a combined web interface that allows to identify disordered protein regions using IUPred2 and disordered binding regions using ANCHOR2.	identification of disordered protein regions from presynaptic proteins	https://iupred2a.elte.hu/
SMART EMBL	SMART (a Simple Modular Architecture Research Tool) allows the identification and annotation of genetically mobile domains and the analysis of domain architectures.	identification of low complexity domains from presynaptic proteins	http://smart.embl-heidelberg.de/
PaxDB	PaxDB is a comprehensive absolute protein abundance database, which contains whole genome protein abundance information across organisms and tissues.	extraction of protein abundance	https://pax-db.org/
STRING	STRING is a database of known and predicted protein–protein interactions. The interactions include direct (physical) and indirect (functional) associations.	extraction of protein–protein interactions between presynaptic proteins	https://string-db.org/cgi/network.pl
Gene Ontology	The Gene Ontology (GO) knowledgebase is the world's largest source of information on the functions of genes. Associations of gene products to GO terms are statements that describe a molecular function, cellular component or biological process	identification of relevant human proteins via their biological process involvement	http://amigo.geneontology.org/amigo

## 3. Results

**Figure 1.**
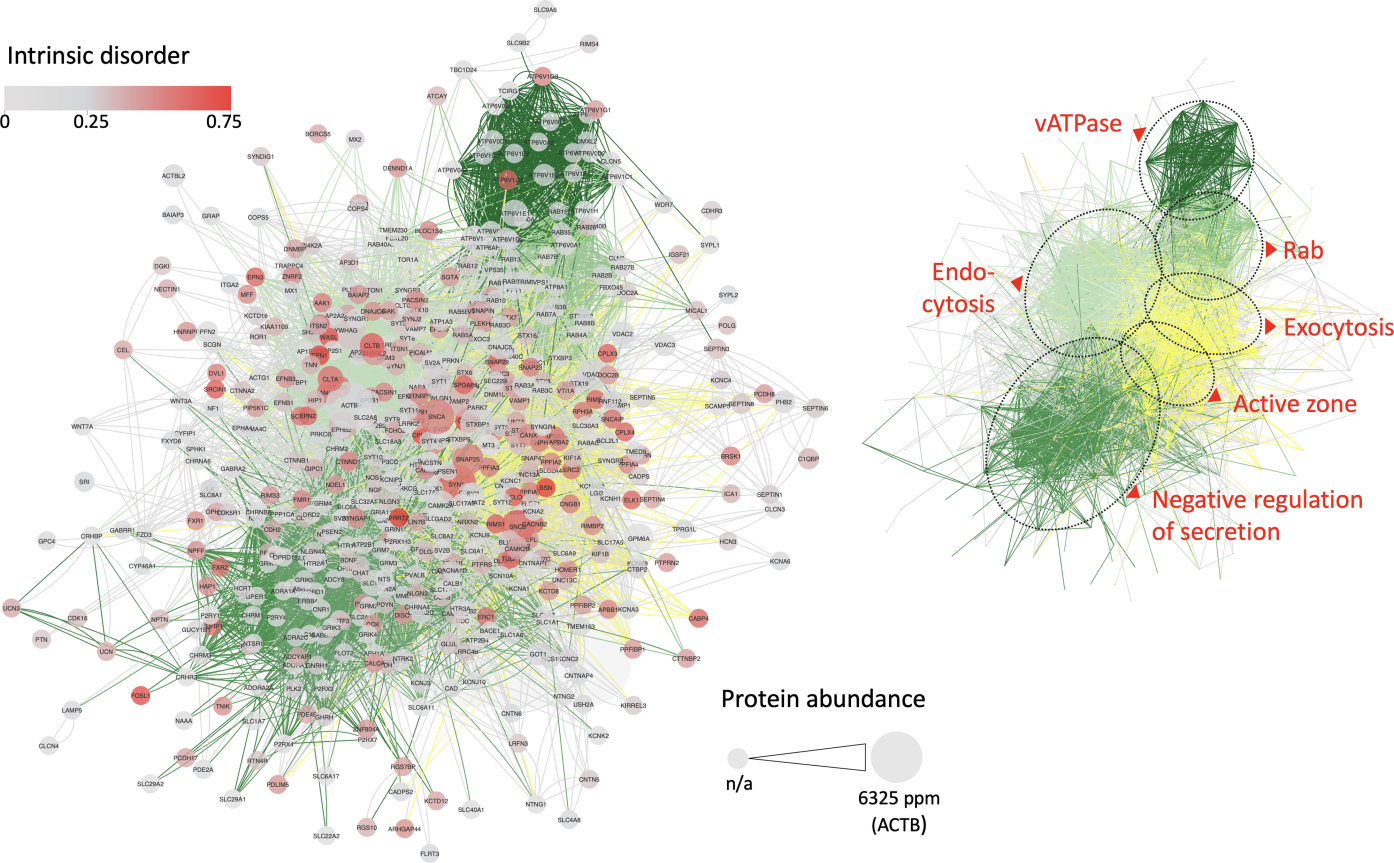
Protein–protein interaction (PPI) network showing six main protein hotspots in the presynapse. PPI network for 578 presynaptic proteins on the left. And small inlet on the right showing the main protein hotspots: active zone, endocytosis, exocytosis, vATPase complex, negative regulation of secretion and Rab proteins. All proteins are represented by their gene names. The node colour (grey to red) represents the average intrinsic disorder of each protein, highlighting high intrinsic disorder groups in red. Protein abundance is encoded by node size to present proteins with high expression and potential relevance as matrix or scaffold proteins. The full size PPI network is available as electronic supplementary material, figure S1.

This study identified 578 presynaptic proteins, for which the PPI network showed six main protein hotspots (figure 1). These were assigned to the following biological processes:

Hotspot 1: Active zone cluster.
Hotspot 2: Endocytosis protein cluster.
Hotspot 3: Exocytosis, priming and fusion protein cluster.
Hotspot 4: Proteins involved in proton transmembrane transport/v-type proton ATPase complex.
Hotspot 5: Two protein clusters involved in the negative regulation of secretion and neurotransmitter reuptake.
Hotspot 6: Rab signal transduction protein cluster.

The prediction data show that Hotspot 1, Hotspot 2 and Hotspot 3 are enriched for proteins with a higher tendency to undergo LLPS. These are interesting hits, since components of the active zone have currently been highlighted to undergo LLPS and first studies demonstrate that LLPS could also play a role in early endocytosis. This article will pick up these examples and put them in context with the findings of this study, giving an overview of recent progress in the field and allowing the systematic view on proteins of the three respective synapse compartments, the active zone, components of clathrin-mediated endocytosis and the hotspot in SV exocytosis. Data for all 578 synaptic proteins on their intrinsic disorder, low-complexity domains and proline content are available as electronic supplementary material, table S1.

## 4. Protein hotspot active zone

**Figure 2.**
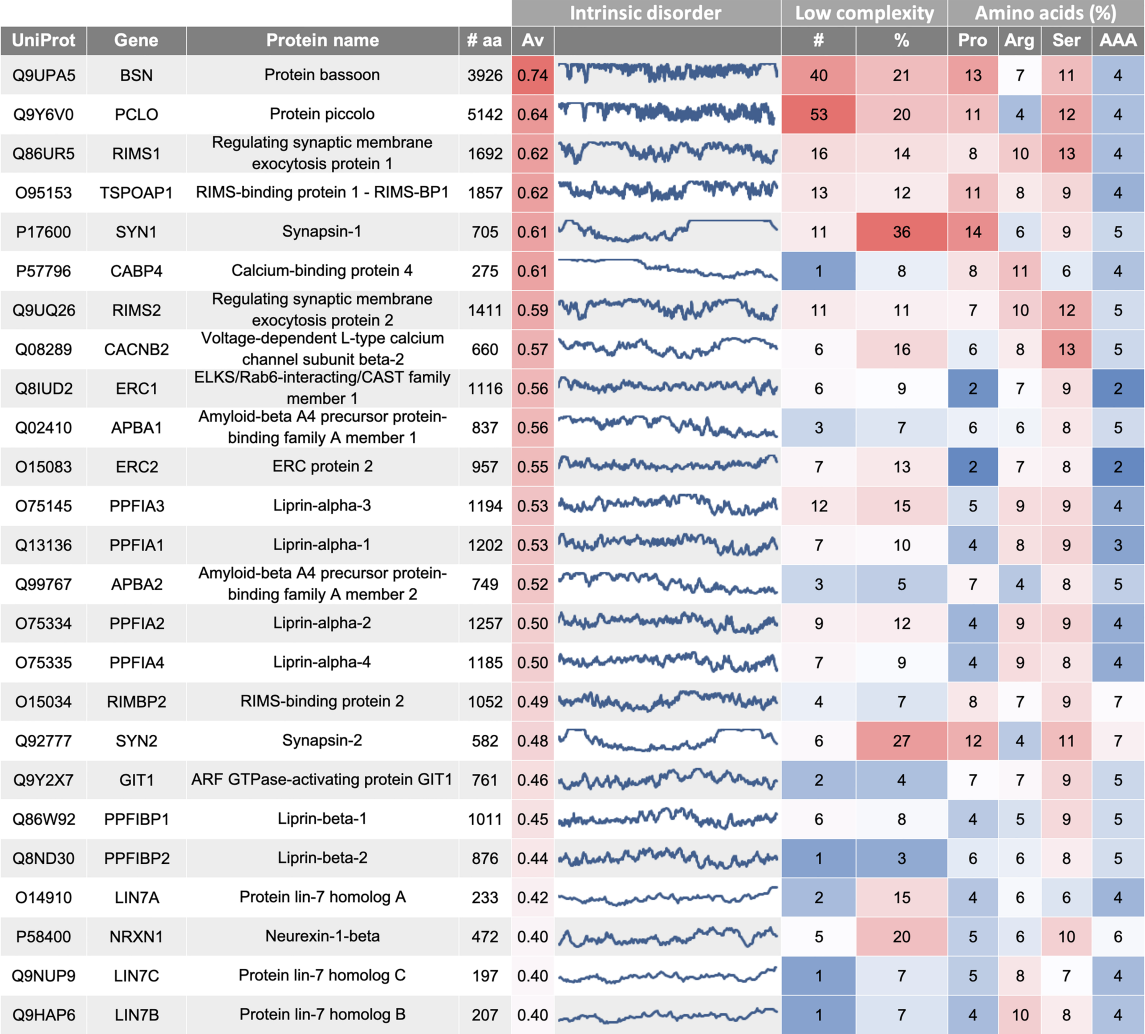
Overview on active zone proteins (excerpt). Protein characteristics include intrinsic disorder (Av, average, line profile along the full protein sequence), low complexity (number of low-complexity domains; percentage of protein length) and percentage of proline (Pro), arginine (Arg), serine (Ser) and aromatic amino acid (AAA) content. This data table is available in full as electronic supplementary material, table S2.

The active zone defines the region of the presynaptic membrane where SV fusion and neurotransmitter release occurs. SV clustering at the active zone seems to occur independently of synapsin-1 [16], and therefore the existence of an additional SV condensate has been proposed early on [17]. This analysis shows that proteins of the active zone hotspot are enriched for high intrinsic disorder, where the three protein groups (i) bassoon/piccolo, (ii) RIM/RIMBP proteins and (iii) liprin proteins are of particular interest (figure 2; electronic supplementary material, table S2).

Bassoon and piccolo, two proteins with high sequence similarity, are the top candidates from the prediction analysis. Both show the largest average intrinsic disorder value of the active zone cluster proteins and also have a high number of low complexity regions, which together span about 20% of the respective protein. Bassoon and piccolo are large scaffolding proteins of the active zone cytomatrix and cover a 100 nm distance from the active zone, as shown by super-resolution microscopy [18]. Simultaneous knock out of both, bassoon and piccolo, leads to a decrease in SV numbers [19], therefore, making them potential candidates for an active zone protein condensate holding the SVs which are not affected by synapsin KO. However, while LLPS has been shown for the protein hits RIM/RIMBP and liprin, no phase separation has been reported for the high-profile candidates' bassoon and piccolo. Therefore, investigation of bassoon/piccolo in relation to their protein partners, i.e. CAST and RIMBP will be of particular interest [20].

RIMS1 (regulating synaptic membrane exocytosis protein 1), which has been shown to undergo LLPS recently [5], was identified as another high intrinsic disorder profile protein. It has been demonstrated that RIMS1 is able to condensate on its own, but phase separation is substantially potentiated in the presence of the RIM-binding protein RIMBP2. All proteins of this class, RIMS1, RIMS2, RIMBP1 and RIMBP2, display a high intrinsic disorder and a relatively high proportion of low complexity. RIM/RIMBP condensates show co-condensation with the intracellular C-terminal region of the voltage-dependent N-type calcium channel subunit alpha-1B (CACNA1). These condensates are, therefore, likely to position voltage-gated calcium channels to the site of SV release [5,21–23]. Upon quadruple knock out of all RIM/RIMBP proteins, the tight tethering and priming of SVs to the active zone is disturbed [24]. However, it seems that overall, the larger pool of SV recruited to the active zone is not severely affected. Here, bassoon and piccolo could come into play, possibly being able to extent and support the RIM/RIMBP condensate. In addition, RIM is also known to bind Rab3, Munc13, ELKS and liprin-alpha proteins [25], which are potential partners adding further functionality to the RIM/RIMBP LLPS condensate.

ELKS and liprin proteins have been identified next, showing high intrinsic disorder and also about 9–15% of low complexity. A very recent paper studying ELKS and SYD-2, the *Caenorhabditi elegans* homologue of liprin-alpha, found that these proteins can undergo LLPS. Different to other LLPS systems, ELKS and SYD-2 matured into solid like structures easily, being less dynamic later in life. Therefore, a role during synapse development is suggested [26]. By contrast, a more dynamic nature is shown for liprin-alpha3, whose phase separation can be reversibly regulated by PKC [27]. Interestingly, ELKS and the liprin proteins show no enrichment for proline, while this is the case for many other synaptic LLPS proteins. For example for synapsin-1, where the proline rich domain is important for its interaction with the SH3 domain protein intersectin [4], and for RIM where proline rich motifs are important for its interaction with RIMBP [5]. Again, also bassoon and piccolo are highly proline rich, with 13% and 11%, respectively.

It was demonstrated recently that synapsin and RIM/RIMBP form separate phases which do not intermix [28]. This further supports the maintenance of two separate SV pools, the reserve pool, where synapsin clusters the majority of SVs, and an SV cluster which is situated in a 100 nm distance of the active zone, regulated by RIM/RIMBP and potential additional proteins. Compared to synapsin-1, all the identified active zone proteins, bassoon/piccolo, RIM/RIMBP, ELKS and liprins, show low abundance levels (all less than 50 ppm, electronic supplementary material, table S1), which speaks for a multicomponent LLPS system, rather than one protein on its own serving as a matrix. Lastly, the calcium binding protein 4 (CABP4) should be highlighted from this study. This protein is involved in neurotransmission at the photoreceptor and shows very high intrinsic disorder values. Furthermore, it has been linked to human rod and cone dysfunction in humans [29]. An overview on all parameters of respective active zone proteins can be found in figure 2 (excerpt) and electronic supplementary material, table S2.

## 5. Protein hotspot clathrin-mediated endocytosis

If the SV cluster and the active zone are regulated and supported by protein LLPS, we might think of synapse LLPS as a more universal mechanism, also involved in other synapse processes. This analysis shows that the endocytosis hotspot is highly enriched for highly intrinsically disordered proteins. Indeed, the disordered regions of many endocytosis proteins have been recognized to regulate membrane curvature via a steric effect [30,31]. A protein region with high intrinsic disorder not folding into a well-defined structure, spans a larger volume than a well-folded region. Therefore, if the protein gets densely packed on the membrane, these regions get close together, creating steric pressure, which is able to bend the underlying membrane. However, it has recently been demonstrated that also protein phase separation contributes to membrane bending [32]. This analysis shows that proteins throughout the endocytosis pathway would have the potential to be involved in LLPS and Eps15/FCho have been reported as a first example in early endocytosis [33].

### 5.1. Early factors of clathrin-mediated endocytosis

**Figure 3.**
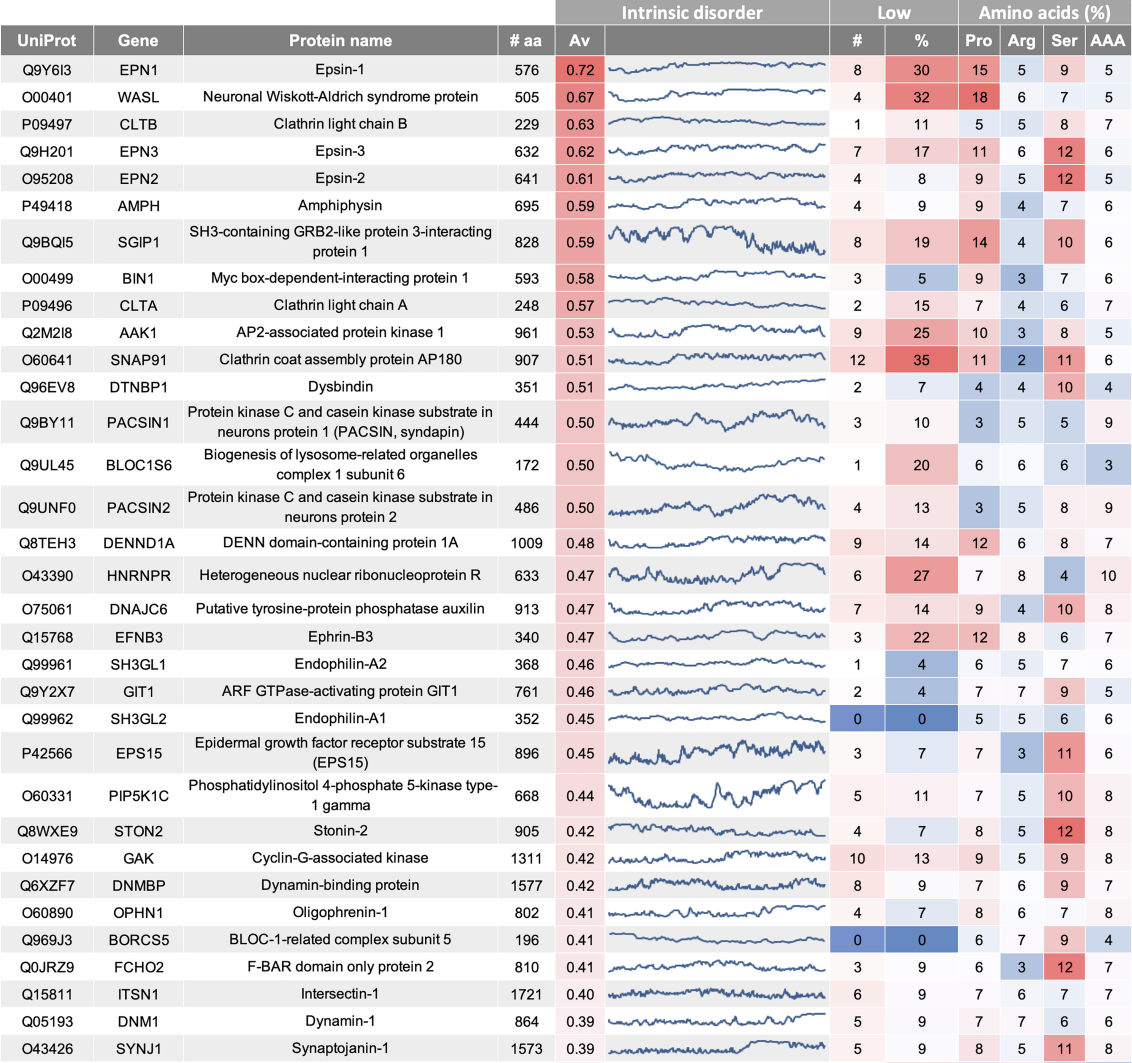
Overview on proteins involved in clathrin-mediated endocytosis (excerpt). Protein characteristics include intrinsic disorder (Av, average, line profile along the full protein sequence), low complexity (number of low complexity domains; percentage of protein length) and percentage of proline (Pro), arginine (Arg), serine (Ser) and aromatic amino acid (AAA) content. This data table is available in full as electronic supplementary material, table S3.

Proteins involved in early steps of the endocytosis process are important to recognize cargo proteins, to induce membrane curvature and to link cargo uptake to the clathrin adaptor AP-2. The most interesting proteins in this analysis are (i) epsin proteins, (ii) Eps15 and FCho, and (iii) Stonin2 and AP180 (figure 3). While many cargo proteins can be recognized by the AP-2 complex directly via conventional tyrosine- or dileucine-based signals, the proteins above have particular roles in SV cargo uptake [34] and are found to support membrane curvature generation.

Epsin-1 is the endocytosis protein with the highest intrinsic disorder in the study, closely followed by epsin-3 and epsin-2. Epsin binds to the lipid membrane via its ENTH (epsin N-terminal homology) domain, while its intrinsic disordered region mediates an effect on membrane curvature [30]. No experimental data on LLPS has been published for epsin proteins to date. However, epsin is known to mediate cargo selection of ubiquitinated proteins, thus LLPS in relation with polyubiquitination might need to be considered [35–37]. Beside a high intrinsic disorder, epsin-1 also has several low complexity regions which together span 30% of the protein, though for epsin-3 and epsin-2 this proportion is lower. All epsin proteins show an enrichment for proline, while epsin-2 and -3 also show a high serine content (12% compared to an average serine content of 7% in the full dataset).

Eps15 (epidermal growth factor receptor substrate 15) and *FCho1/2* (F-BAR domain only protein 1/2) are two additional early endocytosis proteins with high LLPS-tendency. Eps15 and FCho1 have been recently shown to undergo LLPS, where Eps15 forms highly dynamic droplets, which are able to enrich FCho [33]. In addition, it has been shown that this fluid-like property is critical to coordinate the initiation of endocytosis in cells. Proline is not overall enriched in these proteins, but Eps15 is known to have one local proline-rich motif at its C-terminal end, as well as an PQ-rich domain. Interestingly, Ede1, which is the yeast homologue of Eps15, has also been shown to undergo LLPS [38]. Furthermore, it has been demonstrated that other early endocytic proteins lose their punctate localization in the absence of Ede1 [38], which fits well with the current understanding that LLPS is able to specifically co-condense protein partners. As for epsin-1, Eps15 has been shown to modulate membrane curvature [39] and is also involved in ubiquitin cargo selection.

AP180 and Stonin-2, both accessory proteins involved in cargo recognition, exhibit protein regions with high intrinsic disorder. AP180 and the endocytic clathrin adaptor CALM have been shown to be involved in the endocytosis of VAMP SNARE proteins, mediated by the ANTH (AP180-amino-terminal-homology) domain [40,41]. The C-terminal half of AP180, however, shows high intrinsic disorder and has been found to contribute a steric effect on membrane curvature as discussed for epsin-1 [30,42]. AP180 has a particularly high low complexity content of 35% and shows a high percentage of proline and serine (both 11%). Stonin-2 on the other hand is involved in cargo selection of the calcium sensor synaptotagmin [43,44] showing high N-terminal intrinsic disorder and a high serine content (12%). No phase separation has been shown for either, AP180 or stonin-2, however, for stonin-2 an association between several SV proteins has been discussed to facilitate SV protein cargo uptake [45].

The AP2 adaptor complex itself is highly ordered with only very small disordered regions of the alpha and beta-subunits linking its appendage domains. However, the AP2-interacting clathrin-endocytosis protein (KIAA1107) and AP2-associated protein kinase 1 (AAK1) show high intrinsic disorder (figure 3). Also, clathrin heavy chain 1 and 2, building the long triskelia of the clathrin cage, are highly ordered proteins. But again, clathrin light chain B and A are highly disordered with values similar to epsin-2 and -3. They bind to the clathrin heavy chain via a long central a-helix [46,47], while the N-terminal regions accumulate high intrinsic disorder and have been found to support the interaction with other proteins, as for example with the Huntingtin-interacting protein 1-related protein [48–50].

### 5.2. Maturation and fission of the clathrin coated pit

The current analysis shows that also proteins at later steps of endocytosis, involved in maturation and fission of the clathrin coated pit, are enriched for high intrinsic disorder. For this (i) membrane shaping proteins like amphiphysin, endophilins and syndapin, but also (ii) dynamin and (iii) N-WASP, are of particular interest.

Amphiphysin and endophilins are N-BAR domain proteins involved in later stages of endocytosis. Their N-BAR domain folds as a dimer with a concave face, therewith conveying a pressure on the underlying membrane. However, it has been shown that also the intrinsic disordered region substantially contributes to their membrane shaping function by generating steric pressure as found for early membrane shaping proteins [31]. A similar effect could be involved in the membrane shaping behaviour of the F-BAR domain proteins syndapin 1 and 2 (PACSIN1/2), featuring a C-terminal high intrinsic disorder region. Whether protein phase separation of these proteins contributes to membrane bending is not known, but is principally possible as demonstrated for FUS LLPS on lipid vesicles [32].

The functional role of amphiphysin, endophilin and syndapin lies at the periactive zone, however, under resting conditions these proteins localize to the central SV cluster [51]. Here parallels might be drawn to intersectin, which has been demonstrated to localize to the SV cluster at rest, undergoing shuttling to the periactive zone upon synaptic activity [52]. Intriguingly, intersectin is a protein which co-condenses together with synapsin-1 [4], suggesting that intersectin is localized to the SV cluster via LLPS. Once the synapsin condensate disassembles upon synaptic activity, intersectin would be able to diffuse and re-localize. The interaction between intersectin and synapsin-1 is mediated via SH3 domains and the proline rich region of synapsin [4]. Also amphiphysin, endophilins and syndapin constitute each a single C-terminal SH3 domain, which has been shown to weakly interact with synapsin-1 [53]. Thus, a similar mechanism can be imagined for amphiphysin, endophilin and syndapin. Though, if another protein condensate is involved at the periactive zone to capture intersectin and/or other endocytic proteins is speculative. Nevertheless, the phenomenon of endocytosis proteins localizing to the SV cluster might demonstrate a dynamic way able to coordinate the timely delivery of these proteins to the periactive zone upon synaptic stimulation. In addition it has also been discussed that these proteins might contribute to the matrix of the SV cluster [54].

Dynamin-1 is a membrane remodelling GTPases important for vesicle scission. As this, dynamin is a well-structured protein, forming helical polymers around the tubular vesicle neck, mediating membrane constriction [55]. However, it also contains a highly disordered region at its very C-terminal end, which is also enriched in proline (7%, figure 3). This region mediates an interaction with SH3 domain proteins, including amphiphysin, endophilin, syndapin and also intersectin [53,55,56]. Dynamin has not been reported to undergo LLPS, but is also enriched in the SV cluster under resting conditions [51]. Interesting to note, dynamin itself does not have any SH3 domain, only the dynamin-binding protein (DNMBP) contains multiple SH3 domains. Finally, dynamin is a highly abundant protein (greater than 1900 ppm; electronic supplementary material, table S1) and thus might be a candidate as a matrix protein.

N-WASP, the Neural Wiskott–Aldrich syndrome protein, is a well-studied LLPS protein, stimulating Arp2/3-dependent actin nucleation [57,58]. In this analysis N-WASP shows the highest intrinsic disorder after epsin, and has one of the highest proline contents (17.8%). Actin is highly enriched at the periactive zone [52], and a wide range of synaptic proteins, including amphiphysin, DNMBP, syndapin and intersectin, show an interaction with actin or with N-WASP. Furthermore, it has recently been shown that dynamin is regulating actin filament bundling, mediated via its C-terminal proline rich domain [59]. Thus, our understanding of how phase separation could contribute to actin dynamics at the synapse could deliver new insights for synapse organization.

### 5.3. Clathrin coat disassembly

After scission of the clathrin coated vesicle, the clathrin coat itself and adaptor proteins need to be removed. This analysis shows that (i) synaptojanin-1 and (ii) auxilin are proteins with high LLPS tendencies in this pathway.

Synaptojanin-1 is a protein known to couple the fission of clathrin-coated vesicles to the uncoating process [60,61], which is regulated via dephosphorylation of membrane lipids and the release of adaptor proteins [60,62]. Synaptojanin-1 contains a central inositol 5-phosphatase domain and an N-terminal Sac1-like inositol phosphatase involved in PI(4,5)P2 regulation, while its C-terminal domain is highly disordered and enriched in proline. This proline rich region mediates synaptojanin's interaction with SH3 domain proteins, including endophilin, amphiphysin, syndapin and intersectin [63,64]. In this context, endophilin has been demonstrated to regulate the recruitment of synaptojanin's during endocytosis [65,66].

Auxilin (DNAJC6) is involved in the disassembly of the clathrin coat, recruiting the heat shock cognate 71 kDa protein (Hsc70) and serving as Hsc70 cofactor. Hsc70 again is responsible for the actual coat disassembly [67,68]. Auxilin is described to bind very rapidly after the recruitment of dynamin, just during the process of vesicle neck constriction and subsequent fission [69]. Auxilin binding has been reported to be mediated via lipid sensing of its N-terminal PTEN region [69]; however, it also interacts with dynamin. This interaction is specific for the GTP-bound conformation of dynamin, but the GTPase domain nor the GAP domains themselves are sufficient for binding [70], indicating that an additional binding mechanism is involved which has not been described yet.

## 6. Protein hotspot synaptic vesicle exocytosis

As a third hotspot, SV exocytosis proteins were identified. We think of SV exocytosis as a process where protein binding depends on specific protein conformation as for the SNARE complex [71]. But once again intrinsic disorder regions have been discussed to serve a steric function, enabling the proteins to span a higher radius for interaction [72]. Of particular interest regarding LLPS tendencies are (i) complexins, (ii) Rabphilin-3A as well as (iii) the SNARE complex proteins themselves.

**Figure 4.**
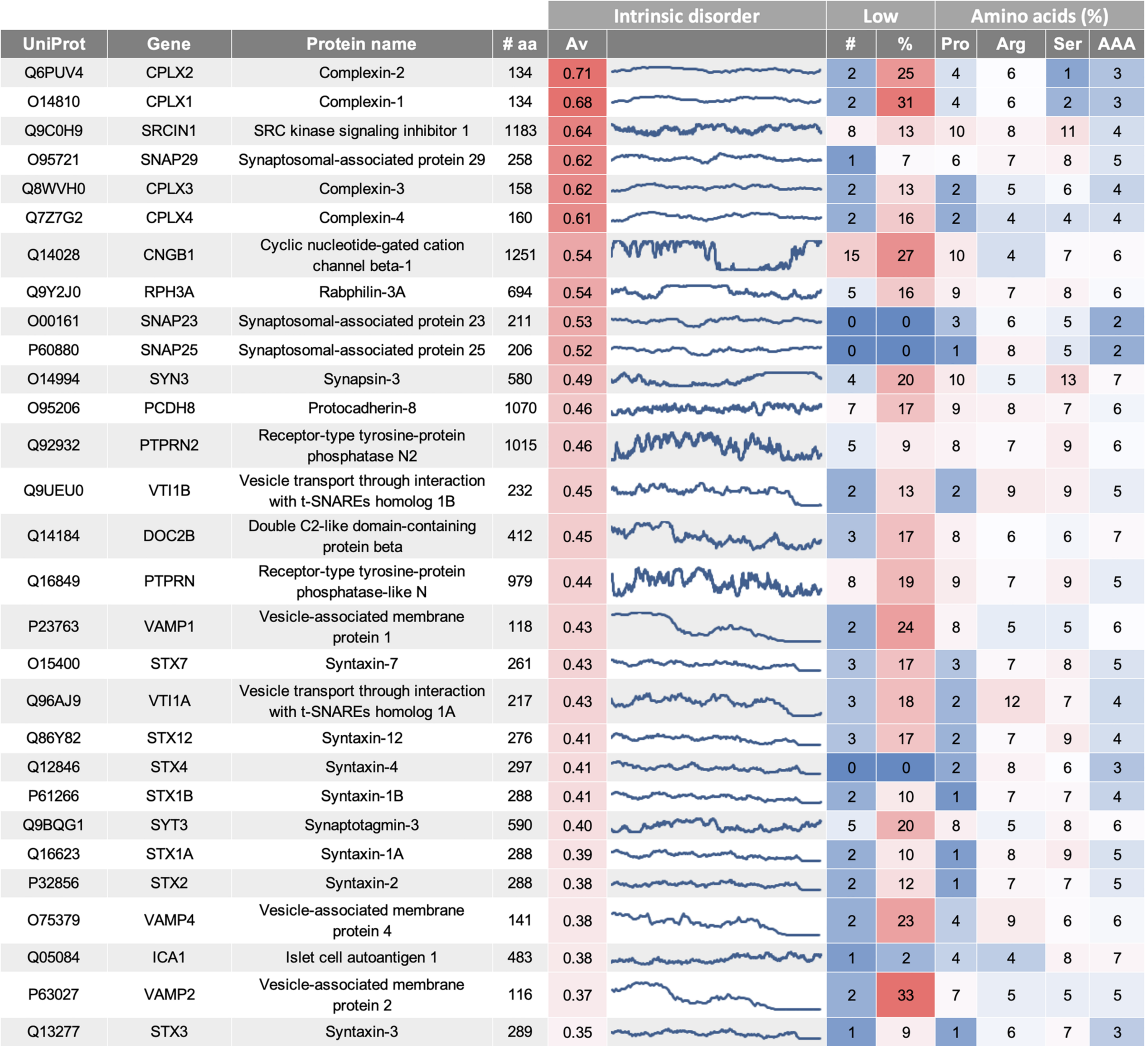
Overview on proteins involved in synaptic vesicle exocytosis (excerpt). Protein characteristics include intrinsic disorder (Av, average, line profile along the full protein sequence), low complexity (number of low complexity domains; percentage of protein length) and percentage of proline (Pro), arginine (Arg), serine (Ser) and aromatic amino acid (AAA) content. This data table is available in full as electronic supplementary material, table S4.

Complexins are small proteins for which inhibitory as well as facilitating effects on SV release have been described. Complexin-2 is enriched in excitatory synapses, while complexin-1 is important for inhibitory synapses. All of the complexins have a high intrinsic disorder and also a high level of low complexity regions (13–31%; figure 4; electronic supplementary material, table S4). However, they are not enriched in proline. Complexins have not been shown to undergo LLPS, but interestingly complexin-2 is one of the highly abundant synapse proteins (greater than 1000 ppm; electronic supplementary material, table S1) and its regulatory role in SV release has remained challenging to explain [73–77].

Rabphilin-3A, which is recruited to the SV membrane via Rab3A also shows a high average intrinsic disorder and is possibly linked to the RIM/RIMBP LLPS systems via Rab3A and Munc13 [25]. Rab3A has been shown to be crucial for the activity-dependent transport of SV to the active zone [78]; therefore, Rab3A and Rabphilin-3A might demonstrate interesting candidates to understand the shuttling between the SV cluster condensate constituted by synapsin-1 and the active zone condensate regulated by RIM/RIMBP.

The three SNARE complex proteins also display high-intrinsic-disorder regions. VAMP2, syntaxin-1A and SNAP25 are well studied for their function in the four helical SNARE complex mediating SV fusion. However, their high intrinsic disorder regions have been discussed to confer an additional role by spanning a higher radius than a folded region would, thereby increasing the likelihood to recruit their binding partners [72]. A role via LLPS has not been shown but could allow the association between SV proteins before complex formation, or on the other hand facilitate protein sorting after exocytosis. The v-SNARE VAMP2 shows the highest low complexity content (33%, N-terminal, electronic supplementary material, table S1) as well as a high proline content (7%). By contrast, the t-SNAREs (i.e. syntaxin-1A and SNAP25) have an extremely low proline content of 3% or below. This might demonstrate a relevant difference since these proteins are throughout localized to the plasma membrane where they are reported to form distinctive nanoclusters [79,80].

## 7. Discussion

Protein phase separation enables proteins to concentrate in a protein dense phase and is evolving as a mechanism relevant for the spatio-temporal organization within cells and the synapse. The concept of LLPS can harmonize current discrepancies, giving a better understanding how proteins are able to accomplish their function. Thus the LLPS model for SV clustering via synapsin-1 is able to overcome certain discrepancies of the classical synapsin-SV-actin tethering model [51]. LLPS can be seen as a mechanism for the regulation of highly dynamic processes and is complementary to the mechanisms we already know. Many of our current findings build upon the fundamental understanding of protein regions and protein interactions known so far. Proteins not only convey one role or mechanism of action, but different properties come together. This we have seen for N-Bar domain proteins regulating membrane curvature by two complementary mechanisms, demonstrating that one mechanism is supported by the other, is serving as a backup or is adding further functionality. The present study, evaluating data on intrinsic disorder, low complexity domains and amino acid composition of synapse proteins, gives an overview of potential LLPS mechanisms at the synapse. It emphasizes how synaptic processes are supported by protein LLPS, opening new question to be asked and stimulating a novel way of thinking on how proteins organize synaptic compartments.

Next steps are the construction of a prediction tool specific to the synapse compartment. Several LLPS prediction tools have been developed over the years including PScore, PLAAC, PSPer, CatGranule, R + Y and LARKS [81,82]. These rely on specific protein properties, like pi–pi contacts, similarity to prion-like domains or FUS-like proteins, or low complexity aromatic-rich segments. Overall these predictions are of course dependent on proteins we already know to undergo protein phase separation and therefore, at the moment, have a high representation of DNA/RNA binding proteins. A new approach solely relies on a prediction due to differences in the amino acid content of LLPS proteins [83]. Again, a bias towards DNA/RNA binding proteins is seen; however, combined with a growing dataset on synapse LLPS this demonstrates an interesting avenue to develop a prediction tool specific for synapse LLPS proteins. In the future, novel technologies in data science including artificial intelligence will allow to develop learning algorithms implementing new data step by step helping us to understand specific and distinct LLPS compartments at the synapse.

## Data accessibility

Data can be accessed via the electronic supplementary material [84].
